# Supplementary material for: Cortical gradient of a human functional similarity network captured by the geometry of cytoarchitectonic organization
Source: Commun Biol. 2022 Oct 30;5:1152. doi: 10.1038/s42003-022-04148-4 (PMC9618576; doi:10.1038/s42003-022-04148-4)
Supplement: Supplementary file 2 — Supplementary information [file 42003_2022_4148_MOESM2_ESM.pdf]

Supplementary Information for

Cortical gradient of a human functional similarity network captured by the geometry of cytoarchitectonic organization

Yao Meng[1,2], Siqi Yang[1,2], Jinming Xiao[1,2], Yaxin Lu[1,2], Jiao Li[1,2], Huafu Chen[1,2], Wei Liao[1,2,*].

***Corresponding authors:** Wei Liao.
Email: weiliao.wl@gmail.com

This PDF file includes:

Supplementary Table 1
Supplementary Figures 1 to 4
Supplementary References

Supplementary Table 1. Correlation between tSNR map with functional metrics

Correlation with tSNR	ALFF	fALFF	Reho	DC	iEfficiency	gEfficiency	Path length
Spearman r	-0.80	0.84	0.06	-0.44	-0.40	-0.41	0.42
p-value$_{SAC}$	< 0.0001	< 0.0001	0.65	0.018	0.023	0.027	0.03

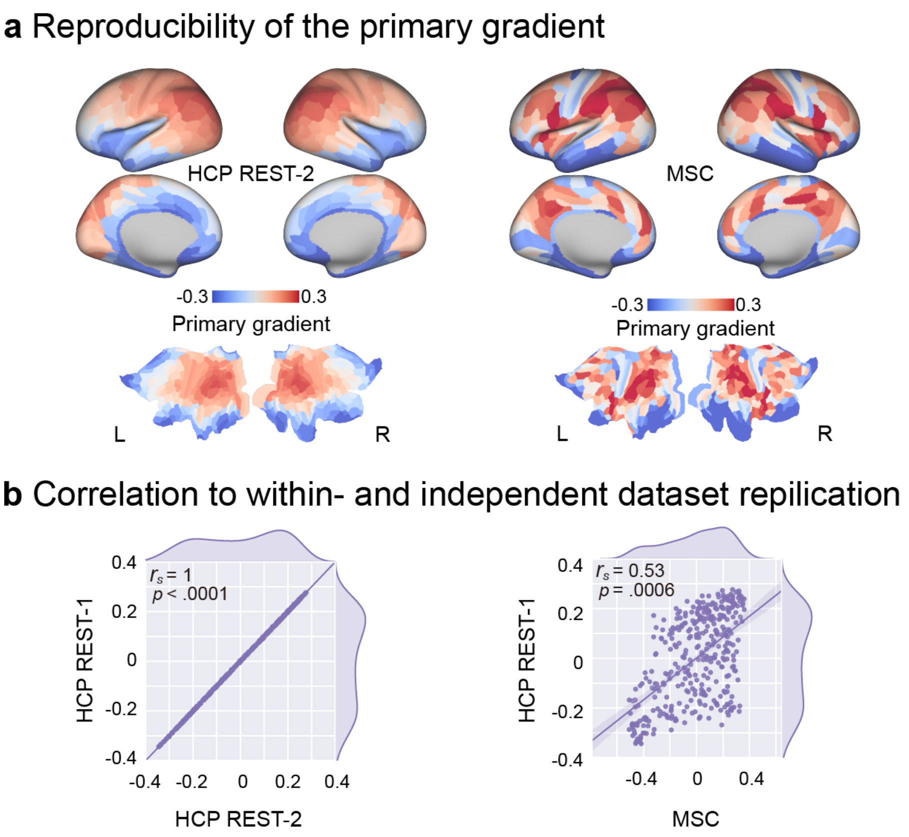

Supplementary Figure 1. The reproducibility of the primary gradient. a The reproducibility of primary gradient has tested in internal validation (HCP REST-2 dataset) and external validation (independent MSC dataset), the primary gradient was rendered on inflated surface and unfolded flat surface. **b** The spatial correlation between reproduced primary gradients with the one in main analysis.

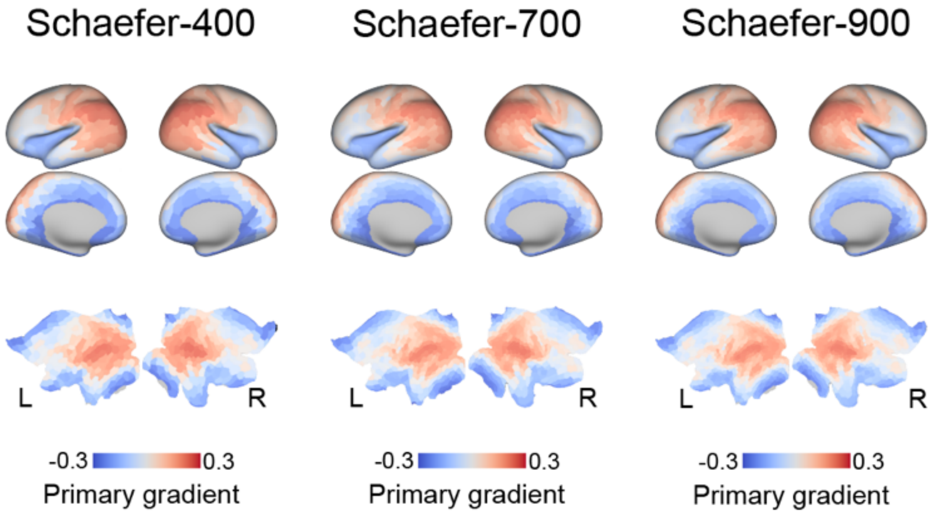

Supplementary Figure 2. The reproducibility of the primary gradient with multiple granularity cortical parcellation schemes. Left panel displays the primary gradient using a relatively identical granularity parcellation (Schaefer-400, Schaefer et al.) [1] to the multi-modal parcellation (MMP) atlas which presented in the main text, middle panel displays the reproduced primary gradient using a relatively identical granularity parcellation (400 parcels). Middle and right panels display other two primary gradient using higher granularity parcellation scheme (700 and 900 parcels, Schaefer et al.) [1].

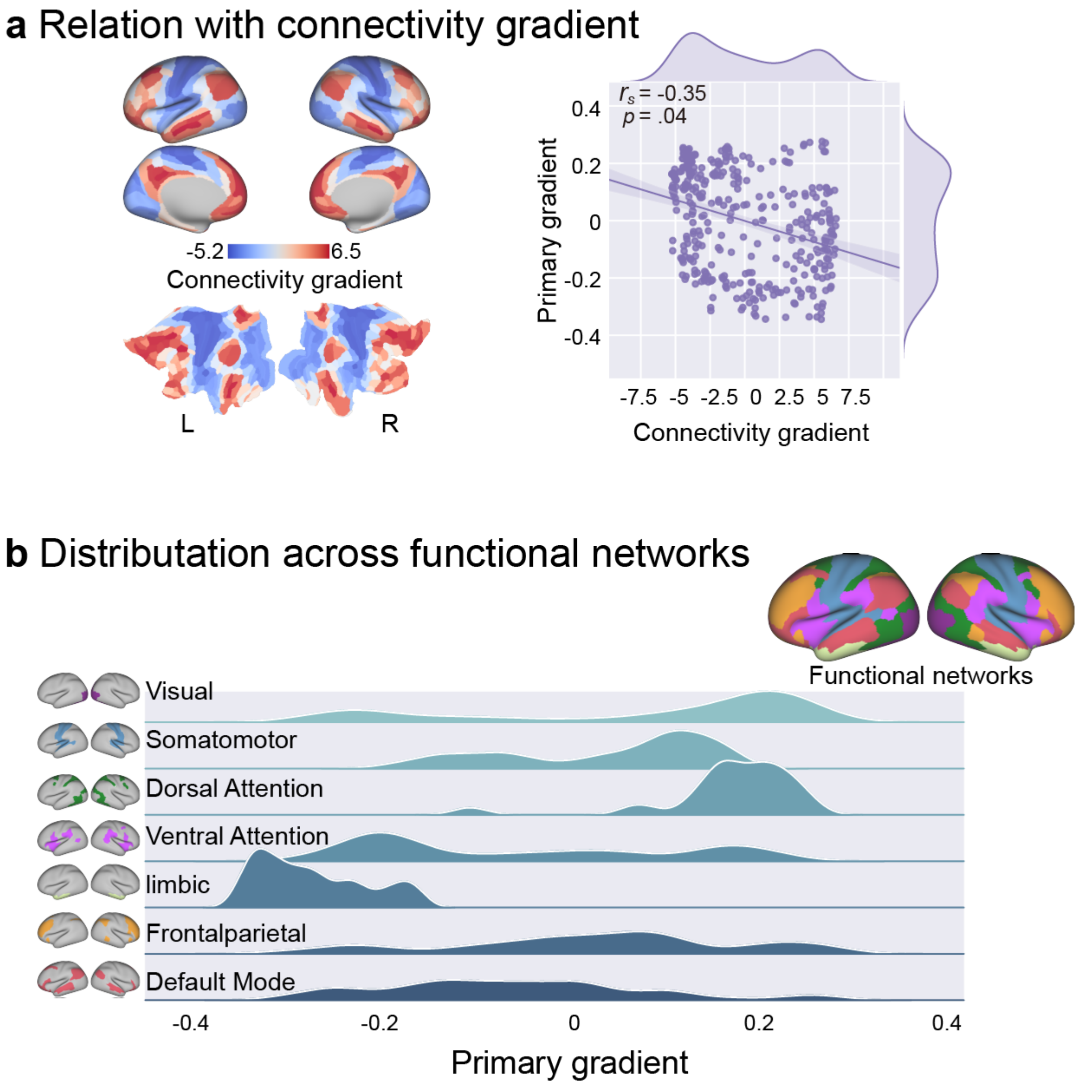

Supplementary Figure 3. Relation with connectivity gradient and the distribution across functional networks. a The connectivity gradient acquired from Margulies et al. [2] (left panel) and the right shows the spatial correlation between the connectivity gradient with the proposed primary gradient (right panel). **b** The distribution of primary gradient across Yeo-7 functional networks (Yeo et al.) [3].

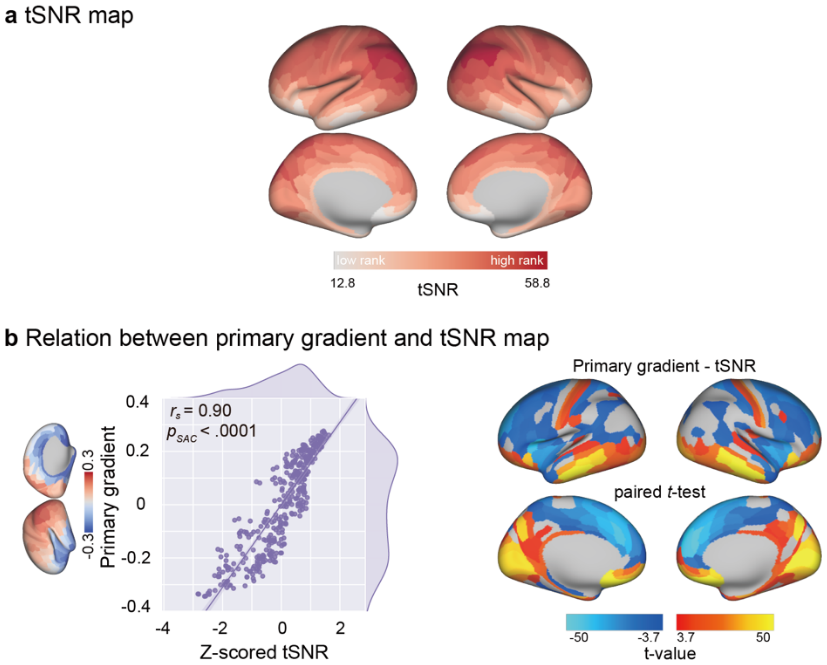

Supplementary Figure 4. Relation with tSNR. a Group-mean tSNR map of HCP REST-1. **b** The correlation and differences (paired *t-test,* Bonferroni corrected) between primary gradient and tSNR maps across HCP-REST1 sample.

Supplementary References

1. Schaefer, A. *et al.* Local-Global Parcellation of the Human Cerebral Cortex from Intrinsic Functional Connectivity MRI. *Cerebral Cortex* 28, 3095–3114 (2018).
2. Margulies, D. S. *et al.* Situating the default-mode network along a principal gradient of macroscale cortical organization. *Proc Natl Acad Sci USA* 113, 12574–12579 (2016).
3. Thomas Yeo, B. T. *et al.* The organization of the human cerebral cortex estimated by intrinsic functional connectivity. *Journal of Neurophysiology* 106, 1125–1165 (2011).
