# Supplementary material for: Cortical gradient of a human functional similarity network captured by the geometry of cytoarchitectonic organization
Source: Commun Biol. 2022 Oct 30;5:1152. doi: 10.1038/s42003-022-04148-4 (PMC9618576; doi:10.1038/s42003-022-04148-4)
Supplement: Supplementary file 5 — Reporting Summary [file 42003_2022_4148_MOESM5_ESM.pdf]

nature portfolio

Corresponding author(s): Wei Liao

Last updated by author(s): 2022/10/08

Reporting Summary

Nature Portfolio wishes to improve the reproducibility of the work that we publish. This form provides structure for consistency and transparency in reporting. For further information on Nature Portfolio policies, see our Editorial Policies and the Editorial Policy Checklist.

Statistics

For all statistical analyses, confirm that the following items are present in the figure legend, table legend, main text, or Methods section.

n/a	Confirmed	
☐	☒	The exact sample size (*n*) for each experimental group/condition, given as a discrete number and unit of measurement
☐	☒	A statement on whether measurements were taken from distinct samples or whether the same sample was measured repeatedly
☐	☒	The statistical test(s) used AND whether they are one- or two-sided<br>*Only common tests should be described solely by name; describe more complex techniques in the Methods section.*
☐	☒	A description of all covariates tested
☐	☒	A description of any assumptions or corrections, such as tests of normality and adjustment for multiple comparisons
☐	☒	A full description of the statistical parameters including central tendency (e.g. means) or other basic estimates (e.g. regression coefficient) AND variation (e.g. standard deviation) or associated estimates of uncertainty (e.g. confidence intervals)
☐	☒	For null hypothesis testing, the test statistic (e.g. *F*, *t*, *r*) with confidence intervals, effect sizes, degrees of freedom and *P* value noted<br>*Give P values as exact values whenever suitable.*
☒	☐	For Bayesian analysis, information on the choice of priors and Markov chain Monte Carlo settings
☒	☐	For hierarchical and complex designs, identification of the appropriate level for tests and full reporting of outcomes
☐	☒	Estimates of effect sizes (e.g. Cohen's *d*, Pearson's *r*), indicating how they were calculated

Our web collection on statistics for biologists contains articles on many of the points above.

Software and code

Policy information about availability of computer code

Data collection	All data analyzed in this manuscript were obtained from the open-access HCP young adults sample (HCP; http:// www.humanconnectome.org/) and Midnight Scan Club (https://openneuro.org/datasets/ds000224/versions/1.0.3).
Data analysis	Original HCP data went through the minimally preprocessing pipeline, aligned to the fs_LR32k group space using Multimodal Surface Matching All area feature-based registration (MSM-All). Data denoising was achieved by FMRIB’s independent component analysis-based X-noiseifier (ICA-FIX). All the custom code used in this research were openly available in https://github.com/YaoMeng94/FSN-Gradient.

For manuscripts utilizing custom algorithms or software that are central to the research but not yet described in published literature, software must be made available to editors and reviewers. We strongly encourage code deposition in a community repository (e.g. GitHub). See the Nature Portfolio guidelines for submitting code & software for further information.

Data

Policy information about availability of data

All manuscripts must include a data availability statement. This statement should provide the following information, where applicable:
- Accession codes, unique identifiers, or web links for publicly available datasets
- A description of any restrictions on data availability
- For clinical datasets or third party data, please ensure that the statement adheres to our policy

All data analyzed in this manuscript were obtained from the open-access HCP young adults sample (HCP; http:// www.humanconnectome.org/) and Midnight Scan

Club (https://openneuro.org/datasets/ds000224/versions/1.0.3). The raw data may not be shared by third parties due to ethics requirements, but can be downloaded directly via the above weblinks. Spearman correlations and confidence intervals were computed using the BrainSMASH toolbox (Burt et al. 2020). Brain map meta-analysis were performed using Neurosynth. All the custom code used in this research were openly available in https://github.com/YaoMeng94/FSN-Gradient.

Human research participants

Policy information about studies involving human research participants and Sex and Gender in Research.

Reporting on sex and gender	Sex effects was not considered in this study.
Population characteristics	Our research sample comprised data from 999 young, healthy adults (female = 541, age = 26.95 ± 3.47 years) from the HCP S1200 release.
Recruitment	The full set of inclusion and exclusion criteria are described previously (Marcus, 2013).
Ethics oversight	As we exclusively used data from the HCP, we relied on their ethics consortium from institutions in the US and Europe, i.e. the 'WU-Minn HCP Consortium'

Note that full information on the approval of the study protocol must also be provided in the manuscript.

Field-specific reporting

Please select the one below that is the best fit for your research. If you are not sure, read the appropriate sections before making your selection.

☒ Life sciences ☐ Behavioural & social sciences ☐ Ecological, evolutionary & environmental sciences

For a reference copy of the document with all sections, see nature.com/documents/nr-reporting-summary-flat.pdf

Life sciences study design

All studies must disclose on these points even when the disclosure is negative.

Sample size	We studied two independent samples from openly-shared neuroimaging repositories, Human Connectome Project and enhanced Midnight Scan Club. Human Connectome Project (HCP; http://www.humanconnectome.org/), comprised data from 999 young, healthy adults (female = 541, age = 26.95 ± 3.47 years) from the HCP S1200 release for whom all four rs-fMRI and structural scans were available. We used Midnight Scan Club (MSC) to test the reproducibility. This sample is openly available from Gordon et al. 2017. This sample consisted of 10 healthy adults completed 12 scanning sessions on 10 sequential days.
Data exclusions	We included participants for whom the MRI images and data had been released (humanconnectome.org) after passing the HCP quality control and assurance standards. The full set of inclusion and exclusion criteria are described previously (Marcus, 2013). Additionally we excluded subjects who failed to complete the scan sessions (less than four resting-state fMRI scan sessions, N = 95) and a batch of incorrectly preprocessed subjects (N = 19) by the HCP.
Replication	We used Midnight Scan Club (MSC) to test the reproducibility. This sample is openly available from Gordon et al. 2017.
Randomization	No randomization is performed.
Blinding	No blinding is done.

Reporting for specific materials, systems and methods

We require information from authors about some types of materials, experimental systems and methods used in many studies. Here, indicate whether each material, system or method listed is relevant to your study. If you are not sure if a list item applies to your research, read the appropriate section before selecting a response.

Materials & experimental systems

n/a	Involved in the study
☒	☐ Antibodies
☒	☐ Eukaryotic cell lines
☒	☐ Palaeontology and archaeology
☒	☐ Animals and other organisms
☒	☐ Clinical data
☒	☐ Dual use research of concern

Methods

n/a	Involved in the study
☒	☐ ChIP-seq
☒	☐ Flow cytometry
☐	☒ MRI-based neuroimaging

Magnetic resonance imaging

Experimental design

Design type: Resting-state fMRI.

Design specifications: not relevant

Behavioral performance measures: HCP: First, to constrain analyses we selected primary markers for cognition, mental and physical health based on the relation of sleep to these traits in HCP. The selected traits include 38 emotional, cognitive, NEO-FFI personality, as well as the 7 PSQI sleep markers for reference, based on the unrestricted phenotypic data as well as 46 mental and physical health markers based on the restricted phenotypic data. For more information on available phenotypes, please see: (https://wiki.humanconnectome.org/display/PublicData).
Inter-individual difference in sleep quality were derived from information of the self-reported Pittsburg Sleep Questionnaire (PSQI) (Buysse, 1989) , which is common measure of sleep quality with significant item-level reliability and validity. For markers of life function, we used BMI (703 * weight/ (height)^2) and the ASR depression DSM-oriented scale for Ages 18-5949 (https://aseba.org/). As a proxy for intelligence we used the NIH Toolbox Cognition Weintraub, 2013 , 'total composite score'.

Acquisition

Imaging type(s): Functional imaging

Field strength: 3 Tesla

Sequence & imaging parameters: Subjects were tested twice in a span of 48 hours, each run is 14.4 minutes (1200 volumes, TR = 0.72)

Area of acquisition: Whole brain scans

Diffusion MRI: ☐ Used ☒ Not used

Preprocessing

Preprocessing software: We used the HCP preprocessed data.

Normalization: Normalization was performed using a single nonlinear transformation according to HCP protocols.

Normalization template: fs_LR_32k

Noise and artifact removal: Data denoising was achieved by FMRIB's independent component analysis-based X-noiseifier (ICA-FIX).

Volume censoring: No volume censoring was performed.

Statistical modeling & inference

Model type and settings: Multivariate functional connectivity and series of deriving metrics. Diffusion map embedding of the similarity between spatial location's functional metrics.

Effect(s) tested: We tested the spatial correspondence between proposed functional gradient (brain map) with multiple cross-scale brain representations using Spearman's correlation coefficient.

Specify type of analysis: ☐ Whole brain ☒ ROI-based ☐ Both

Anatomical location(s): We used HCP MMP atlas and Schaefer atlas (400, 700, 900).

Statistic type for inference (See Eklund et al. 2016): Parcel-wise.

Correction

Significance were determined by comparing empirical correlation values with spatial autocorrelation spatial-autocorrelation accounted null model.

Models & analysis

n/a	Involved in the study
☐	☒ Functional and/or effective connectivity
☐	☒ Graph analysis
☐	☒ Multivariate modeling or predictive analysis

Functional and/or effective connectivity	Pearson correlation
Graph analysis	Using a weighted graph of the functional connectivity, we calculated node degree, efficiency clustering coefficient.
Multivariate modeling and predictive analysis	We used diffusion map embedding to conduct the dimension reduction.
